# Experimental and Statistical Evaluation of Infill Density Effects on the Mechanical Properties of FDM-Printed PLA, ABS, PC, and PETG Engineering Materials

**DOI:** 10.3390/polym18161998

**Published:** 2026-08-17

**Authors:** Dilşad Akgümüş

**Affiliations:** Engineering Faculty, Department of Mechanical Engineering, Istanbul Aydin University, 34295 Istanbul, Turkey; dilsadakgumus@aydin.edu.tr

**Keywords:** additive manufacturing, fused deposition modeling, infill density, thermoplastic polymer

## Abstract

Additive manufacturing (AM) has become prominent among advanced manufacturing technologies in recent years due to the advantages it offers in design flexibility, low material waste, and digitalization of the manufacturing process in cases where complex geometries are difficult or costly to produce with traditional methods. Fused deposition modeling (FDM) technology, which is based on the modeling of thermoplastic polymers by melting them layer by layer in a controlled manner, has become widespread in both industrial and academic applications due to its simplicity of process, low equipment cost, wide material compatibility, and sustainable manufacturing approach, and is effectively used in many areas from rapid prototyping to functional final products. In this study, the mechanical performances of different engineering polymers (ABS, PC, PLA, and PETG) produced with the FDM method at 30% and 60% infill densities were evaluated comparatively. The produced specimens were subjected to tensile, Charpy impact, hardness, and SEM analyses; structural strength and microstructure properties of the materials were examined. The results revealed that each material exhibited unique mechanical properties suitable for different applications. ABS was found suitable for applications requiring energy absorption with its superior impact absorption capacity and ductility. PC attracted attention with its highest strength and rigidity values, indicating that it can be preferred in structural parts where mechanical strength is critical. While PLA, one of the sustainable materials, stands out for biomedical components with its high ductility and flexibility properties, PETG provides advantages for carrier structures thanks to its better impact resistance and dimensional stability. The findings reveal that FDM technology can optimize mechanical properties through parameters such as material type and infill density, thereby providing a useful basis for material selection and design optimization in engineering applications.

## 1. Introduction

Additive manufacturing (AM) is an advanced manufacturing technology based on printing time, unlike traditional machining methods. AM technologies are widely used in sectors requiring high precision and performance such as aviation, space, automotive, defense, biomedical, and implant production. A wide range of engineering materials such as thermoplastic and thermoset polymers, metals (titanium, aluminum, stainless steel, etc.), ceramics, and various nanocomposites can be used in production with this technology [1,2].

Fused deposition modeling (FDM) is one of the most widely used methods among polymer-based additive manufacturing technologies and involves the deposition of thermoplastic filaments, which are heated and made semi-fluid during the production process, onto the surface in layers via a nozzle. In the FDM technique, production quality is directly related to parameters such as printing temperature, build platform temperature, layer thickness, fill rate, printing orientation, and printing speed. The appropriate optimization of these parameters plays a decisive role in the mechanical properties, surface roughness, dimensional accuracy, and internal structural integrity of the parts produced [3,4].

A wide variety of thermoplastic polymers can be used in FDM-based 3D printing technologies for different applications; among them, engineering materials such as acrylonitrile butadiene styrene (ABS), polycarbonate (PC), polylactic acid (PLA), polyethylene terephthalate glycol (PETG), polyamide (PA), and polyether ether ketone (PEEK) are particularly prominent. In this study, the four most commonly used thermoplastic polymers were examined. ABS is an engineering thermoplastic composed of acrylonitrile, butadiene, and styrene. It is widely used in FDM applications because of its good mechanical strength, high impact resistance, and excellent chemical resistance to acids and alkalis. In addition, ABS exhibits good dimensional stability and can be processed at recommended printing temperatures ranging from 210 to 270 °C [1]. PC filament is a high-performance engineering thermoplastic widely used in FDM applications due to its high strength, dimensional stability, and good resistance to heat and impact. With these properties, polycarbonate is widely used in demanding engineering applications, including protective equipment, transparent safety components, and impact-resistant structures [4]. PLA is a linear aliphatic thermoplastic polyester derived from lactic acid obtained from renewable resources such as corn starch and sugarcane. Due to its biodegradability, good mechanical strength, high stiffness, and ease of processing, PLA has become one of the most widely used materials in FDM applications [4,5]. PETG is produced by modifying polyethylene terephthalate (PET) with glycol during polymerization, where the letter “G” defines glycol modification. Compared with PET, PETG exhibits lower brittleness, improved toughness, and enhanced processability. In addition, PETG offers good dimensional stability, reduced warpage during FDM printing, and is a recyclable thermoplastic, making it a suitable material for engineering and functional applications [2].

In recent years, studies have been conducted to investigate the mechanical behavior of thermoplastic polymers used in the FDM technique, focusing on the effects of various process parameters such as infill density, layer thickness, and reinforcement techniques. Gunasekaran et al. studied PLA material wire filament with the FDM technique; specimens were created under optimum process parameters and by changing the infill density. The infill density of PLA printed specimens was changed to 25%, 50%, 75%, and 100%. It was determined that the specimens printed with 100% infill density reflected better mechanical properties and showed better mechanical properties with increasing infill density [5]. In their study, Vardhan et al. investigated the tensile properties of metal sputtered thermoplastic components and their possible interactions with other process parameters such as infill density and bed temperature of the FDM printer using Taguchi and ANOVA techniques. The composites experienced higher tensile strength compared to both raw PLA components and aluminum spray-coated FDM parts. The study shows that the tensile properties are significantly affected by the infill density and the number of stacked metal spray layers; the peak strength is directly proportional to the infill density while inversely proportional to the number of layers [6]. Agrawal et al. investigated the combined effect of infill pattern, density, and layer thickness on the mechanical properties of 3D-printed ABS parts. They highlighted that concentric infill patterns at 80% density and a layer thickness of 100 μm yielded the best mechanical properties. Their study emphasizes the importance of optimizing these parameters to achieve superior performance in FFF-based manufacturing [7]. Sedlak et al. explored the impact of environmental degradation factors such as UV radiation, high temperature, and humidity on the mechanical properties of 3D-printed PLA, PETG, ABS, and ASA specimens. They found that PLA showed significant degradation under UV exposure, while ABS and ASA exhibited higher durability under extreme conditions [8]. Szust and Adamski investigated the effects of process parameters (printing direction and layer height), the thermal annealing process, and the re-salting process on the tensile properties and anisotropy of FDM parts. The tensile strength of PETG parts produced with FDM measured in the Z direction increases above 300% after the re-salting process, and the anisotropy of the parts becomes negligible [9]. Balasrinivasan et al. performed a comparative analysis of PLA, PETG, and their carbon fiber reinforced composites. The study concluded that carbon fiber-reinforced PLA and PETG exhibited superior mechanical properties compared to their non-reinforced counterparts. The reinforcement increased tensile strength and reduced deformation under load [10].

Recently, PETG/PLA blends have attracted considerable attention for enhancing the mechanical performance of FDM-printed components. It has been reported that the mechanical properties of these blends strongly depend on their composition, with PLA-rich blends exhibiting higher tensile strength and PETG-rich blends providing improved toughness. Moreover, SEM analyses revealed that interlayer bonding and phase morphology play a key role in determining the mechanical performance of the printed parts [11]. Kadhum et al. studied the effect of various infill patterns on the mechanical and surface properties of 3D-printed parts. They found that honeycomb and triangular patterns provided a balance between weight reduction and structural strength, making them suitable for lightweight applications [12]. Kumar et al. conducted an analysis to determine the mechanical strength of carbon fiber-reinforced PETG thermoplastics and optimize various machine parameters. The results of tensile, flexural, and hardness tests were optimized using a Taguchi L9 experimental approach. The optimum tensile and flexural strength and hardness obtained in this research are 31.567 MPa, 35.045 MPa, and 67.0011 BHN, respectively. The most desirable machine parameters revealed in this research are a printing speed of 60 mm/s, infill density of 80%, and layer height of 0.2 mm. Optimized values for machine parameters have been observed to be useful in selecting working ranges for 3D printing PETG material. It was observed that the strength of PETG increased further when reinforced with carbon fiber [13].

Zorlu examined the recycling of PC/ABS raw material, which is used quite frequently in the automotive industry. During production, the products separated as scrap were crushed and made suitable for the granule drawing process. The crushed materials were granulated by being pulled in special injection machines and mixed with pure raw materials at a weight ratio of 5% and 10% to produce the final semi-finished product used in the automotive industry. The obtained parts were subjected to free-free vibration tests, and it was observed that as the crushing rate increased, the material became more brittle in the 1st mode, and in the 2nd mode, the material tended to soften [14]. Gultekin examined the molding parameters of recycled acrylonitrile butadiene styrene (ABS) plastic material in the study. Recycled ABS material with metal additives provides a positive effect thermal conductivity and electrical conductivity properties have a positive effect, while the mechanical property and fluidity have a negative effect [15]. Martins et al. evaluated the tensile, fatigue, and microstructural behavior of FDM-printed PLA and PETG materials. PLA showed higher tensile strength (55 MPa) and stiffness than PETG, whereas PETG exhibited higher ductility. SEM images revealed interlayer bonding, air voids, and fatigue crack propagation features, while XCT analysis measured porosity values of 9.3% for PLA and 12% for PETG, demonstrating the influence of internal defects on mechanical performance [16]. Menargues et al. investigated the influence of FDM processing parameters on the tensile strength of PLA, ABS, and PETG materials. They reported that increasing infill density and wall number significantly improved tensile strength, with PLA increasing by 1173 N between 20% and 80% infill density and PETG reaching 1207 N with five walls. Conversely, increasing printing speed reduced the tensile strength of PLA by 13%, while the effect of extrusion temperature varied depending on the material [17]. Rao et al. evaluated the influence of filament material (PLA, PETG, and ABS) and FDM process parameters on the mechanical performance of printed parts. They reported that PLA achieved the highest tensile strength (39.2 MPa) and hardness, whereas PETG exhibited the greatest elongation and flexural strength.

Moreover, ANOVA results indicated that material type was the most significant factor affecting tensile strength (66.7% contribution), while infill pattern and infill density were the dominant factors influencing elongation [18]. Nassar et al. evaluated the influence of infill density, infill pattern, material type (PLA and CF-PLA), and thermal annealing on the mechanical behavior of FDM-printed polymers. They reported that thermal annealing improved tensile strength and hardness by enhancing interlayer adhesion and reducing porosity, while elastic modulus was not significantly affected. In addition, material type and infill density were identified as the most influential factors governing mechanical performance, with stronger interaction effects observed after heat treatment [19]. Kafil et al. investigated the tensile behavior of a novel PETG/ABS/ASA blended filament fabricated by FDM using Response Surface Methodology (RSM). They reported that layer thickness and infill density were the dominant parameters governing tensile strength, whereas print speed had only a minor influence. In addition, ANOVA confirmed significant quadratic and interaction effects, and the developed predictive model (R^2^ = 0.9931) demonstrated that thin layers combined with high infill density provided the highest tensile strength [20].

This study, unlike the studies in the literature that generally examine the effects of infill density or other FDM parameters on a single material type, comparatively analyzes the mechanical and microstructural properties of four different engineering thermoplastic materials (ABS, PLA, PETG, and PC) produced under the same production conditions with two different infill densities (30% and 60%). All specimens were produced under identical ambient conditions (room temperature and relative humidity), while processing parameters (the nozzle and build plate temperatures) differed for each material and were adjusted according to the manufacturer’s recommended processing conditions for each material. The findings provide an experimental basis for optimizing material selection and infill density in FDM processes and serve as a lead study for engineering applications requiring enhanced mechanical performance.

## 2. Materials and Methods

In this study, test specimens were produced from ABS, PC, PLA, and PETG filaments at 30% and 60% infill density using a FDM type 3D printer (GEMA brand AFZ 227 model 3D printer (Istanbul, Turkey)) to investigate their mechanical and microstructural properties. ABS, PC, PLA, and PETG filaments were supplied by Porima in 1.75 mm diameters. The physical and mechanical properties of the filaments used in this study, as provided by the manufacturer (Porima, Yalova, Turkey), are presented in Table 1.

The Ultimaker Cura 5.6.0 program was used to prepare the G-code of the specimens before printing on the 3D printer, and the raft structure was used to ensure better adhesion of the print to the surface. The specimens were printed using a grid infill pattern with a layer height of 0.2 mm. No support was needed due to the placement position of the parts. All specimens were grouped according to their infill densities (30% and 60%) and printed on a GEMA brand AFZ 227 model 3D printer (Istanbul, Turkey) in 10 h. The test specimens were ready after the burrs and roughness on the printed specimens were cleaned. The FDM process parameters used in this study are presented in Table 2.

**Figure 1:**
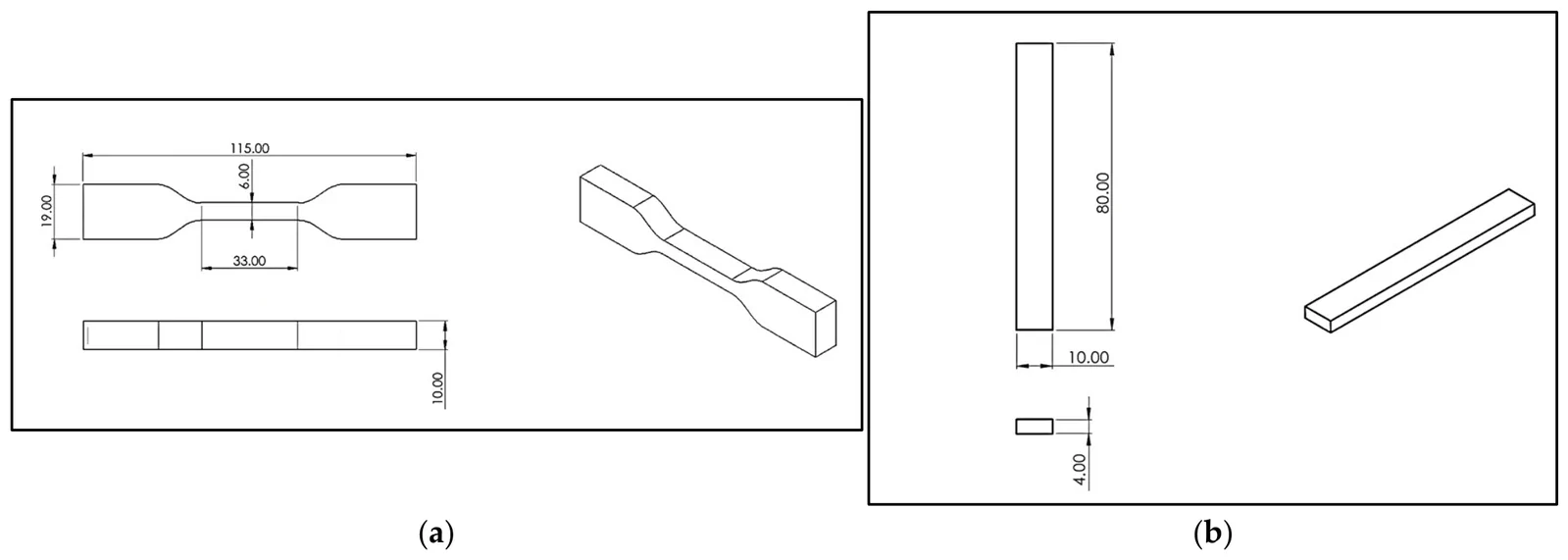
Technical drawing of (**a**) tensile test specimen, (**b**) Charpy impact test specimen.

Hardness, tensile, and Charpy impact tests were performed to determine the mechanical properties of the specimens, and SEM-EDS analysis was performed to determine the microstructure properties. A technical drawing of tensile test specimens is given in Figure 1a according to the ASTM D638-4 standard, and that of the Charpy impact test is given in Figure 1b according to the ISO 179 standard. The Shore D hardness measurements were performed according to ASTM D2240 standard. In addition, three specimens of each polymer material with dimensions of 10 × 10 mm were produced to be used in hardness test and SEM-EDS analysis.

**Figure 2:**
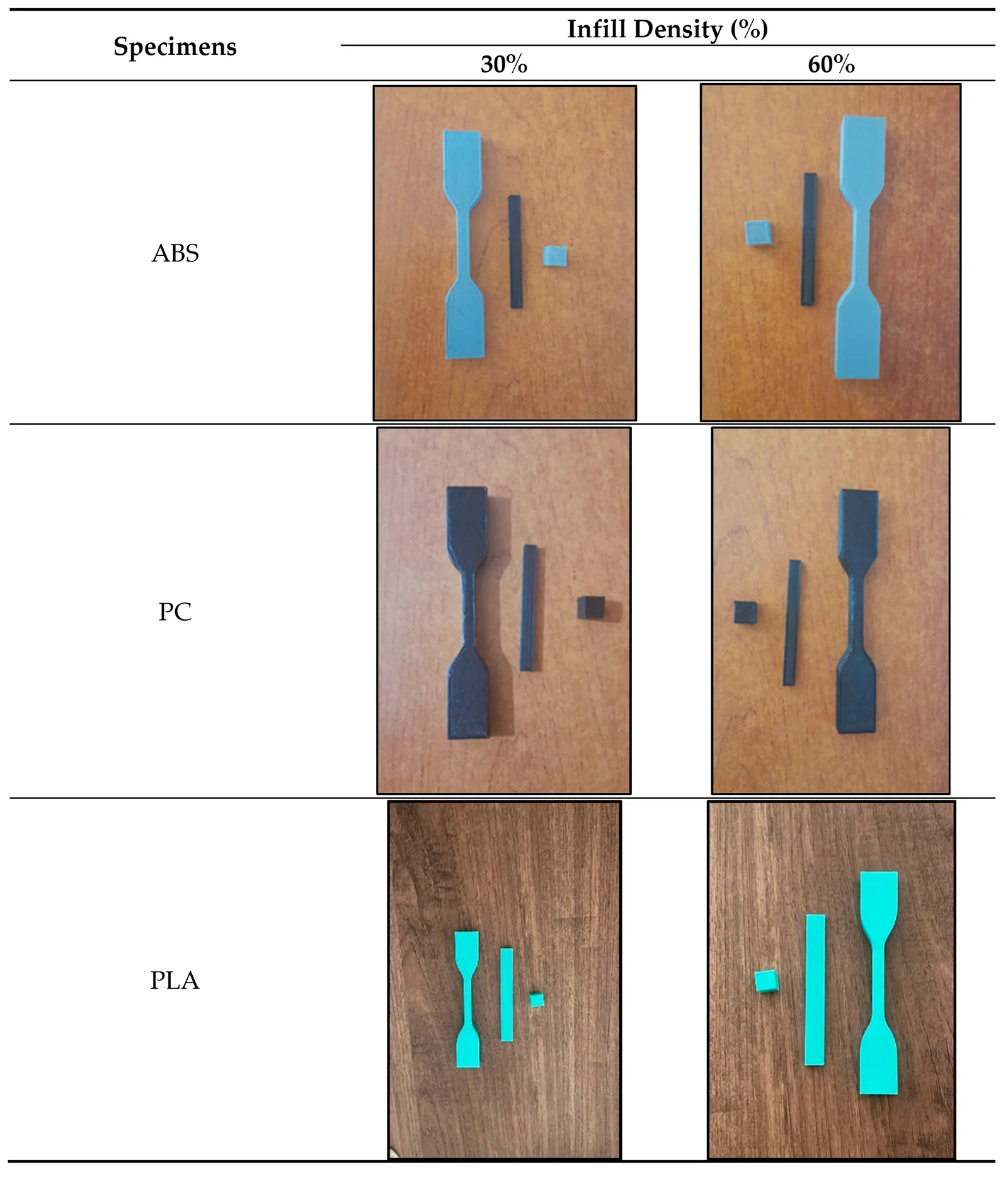

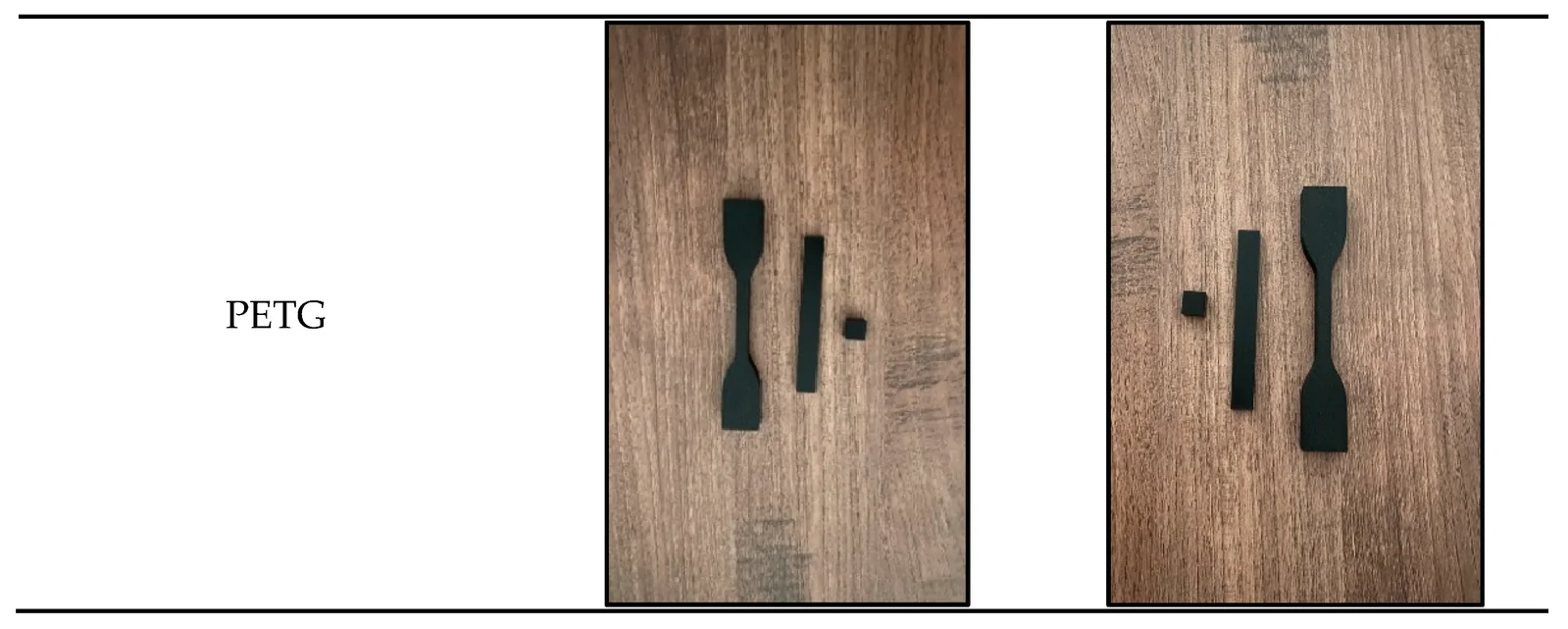
Test specimens.

All specimens produced at 30% and 60% infill density are shown in Figure 2.

The hardness test was performed using a Type D Durometer (Pepisky, Shenzhen, China) device by applying a load in the range of 0–44.5 N. For the hardness measurement, the specimens were placed on a flat surface, five measurements were taken from each specimen surface, and the average hardness values were determined in Shore D. The tensile test was performed on a Mohr & Federhaff brand 7079 model (Mannheim, Germany) 100 kN load capacity tensile machine. The specimens were fixed by placing them in the appropriate position between the jaws of the machine, and the tensile force was applied to the fixed specimens at a speed of 5 mm/min. The Charpy impact tests were performed using a Zwick/Roell brand RKP300 model (Ulm, Germany) impact testing machine equipped with a standard hammer (energy of 2 J). The specimens were positioned horizontally on the test support and subjected to a pendulum impact at room temperature. Each test was repeated three times for accuracy, and the average absorbed energy values were recorded in joules (J). The microstructures and quantitative elemental analysis of the specimens were examined using a ZEISS brand EVO LS10 model SEM-EDS device (Oberkochen, Germany). Before the analysis, the test specimens were placed in holders called stubs and coated with gold Palladium in a Quorum brand SC7620 Sputter Coater model device (East Sussex, UK) to provide conductivity. Argon gas was used in the coating process, and it took an average of 120 s at 24 × 10^−2^ mm rods and 10 milliamperes of current. Standard deviation values for hardness, tensile, and impact tests were also determined to assess the consistency and repeatability of the measurements. Two-way and multifactorial analysis of variance (ANOVA) tests were applied to further assess the statistical significance of the observed differences between different material types and infill densities.

## 3. Results

### 3.1. The Hardness Test

**Figure 3:**
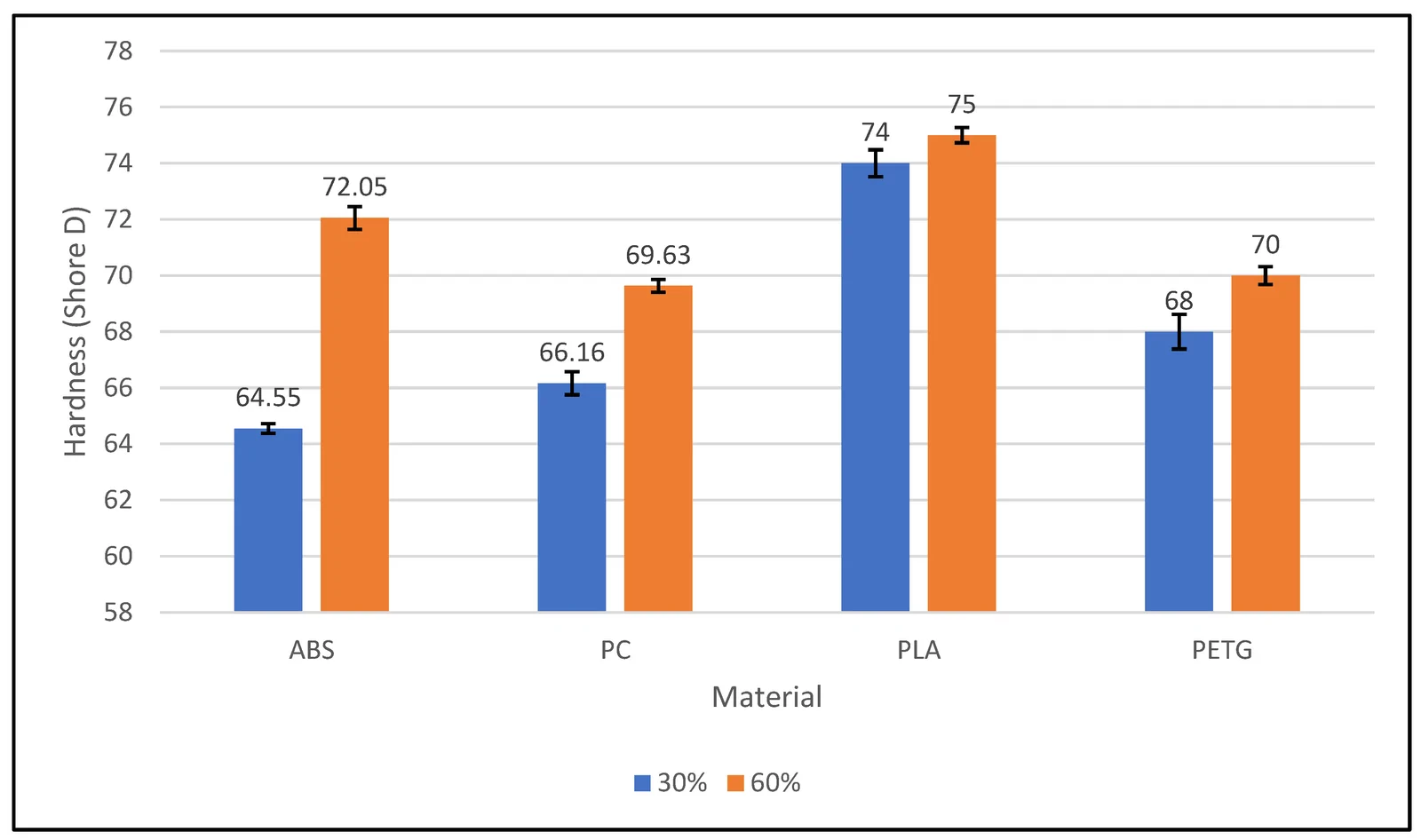
The graph of the hardness test.

Shore D hardness values of specimens produced with different infill densities are presented in Table 3 and Figure 3. Measurements were taken from five different regions of each specimen, and the average hardness, standard deviation, and coefficient of variation values were determined.

For the hardness test, the standard deviation (0.17–0.62 Shore D) and coefficient of variation (0.26–0.91%) values indicate low data dispersion and high repeatability. The low standard deviation values show that the measurements were closely clustered around the mean, while all coefficient of variation values below 1% confirm high measurement precision and reliability. According to the results, an increase in hardness values was observed for all materials with increasing infill density. This was attributed to the increased material density and decreased porosity in the 60% infill density specimens, along with increased surface resistance to indentation.

Among the tested materials, PLA exhibited the highest hardness values at both 30% and 60% infill densities, with Shore D values of 74 and 75, respectively. This result highlights the inherently rigid molecular structure and high surface hardness of PLA, indicating its suitability for applications where surface hardness is a critical parameter. PETG specimens followed PLA, with Shore D values of 68 and 70, suggesting a balanced combination of flexibility and hardness. PC specimens exhibited lower hardness values in comparison to PLA and PETG, measuring 66.16 at 30% infill and 69.63 at 60% infill. Although PC is known for its high strength and thermal resistance, it demonstrates a more ductile behavior under Shore D hardness testing conditions. ABS specimens recorded the lowest hardness values among all materials tested, increasing from 64.55 at 30% infill to 72.05 at 60% infill. This outcome indicates that ABS possesses a more brittle nature compared to PC, which is consistent with its high energy absorption capacity observed in impact tests. Comparative analysis revealed that PLA is more brittle than PETG, while ABS exhibits greater brittleness relative to PC.

Two-way ANOVA analysis in Table 4 revealed that both material type and infill density had statistically significant effects on the Shore D hardness values of FDM-printed specimens (*p* < 0.001), indicating that their effects were significant at the 95% confidence level. Among the examined factors, material type stood out as the most dominant variable on hardness, explaining 59.34% of the total variance, while infill density ranked second with 27.87%. Furthermore, a statistically significant interaction effect (*p* < 0.001) was observed between material type and infill density, demonstrating that the effect of infill density on hardness varies depending on the polymer type used. The error variance was limited to only 1.28%, demonstrating high experimental consistency and model suitability.

### 3.2. The Tensile Test

**Figure 4:**
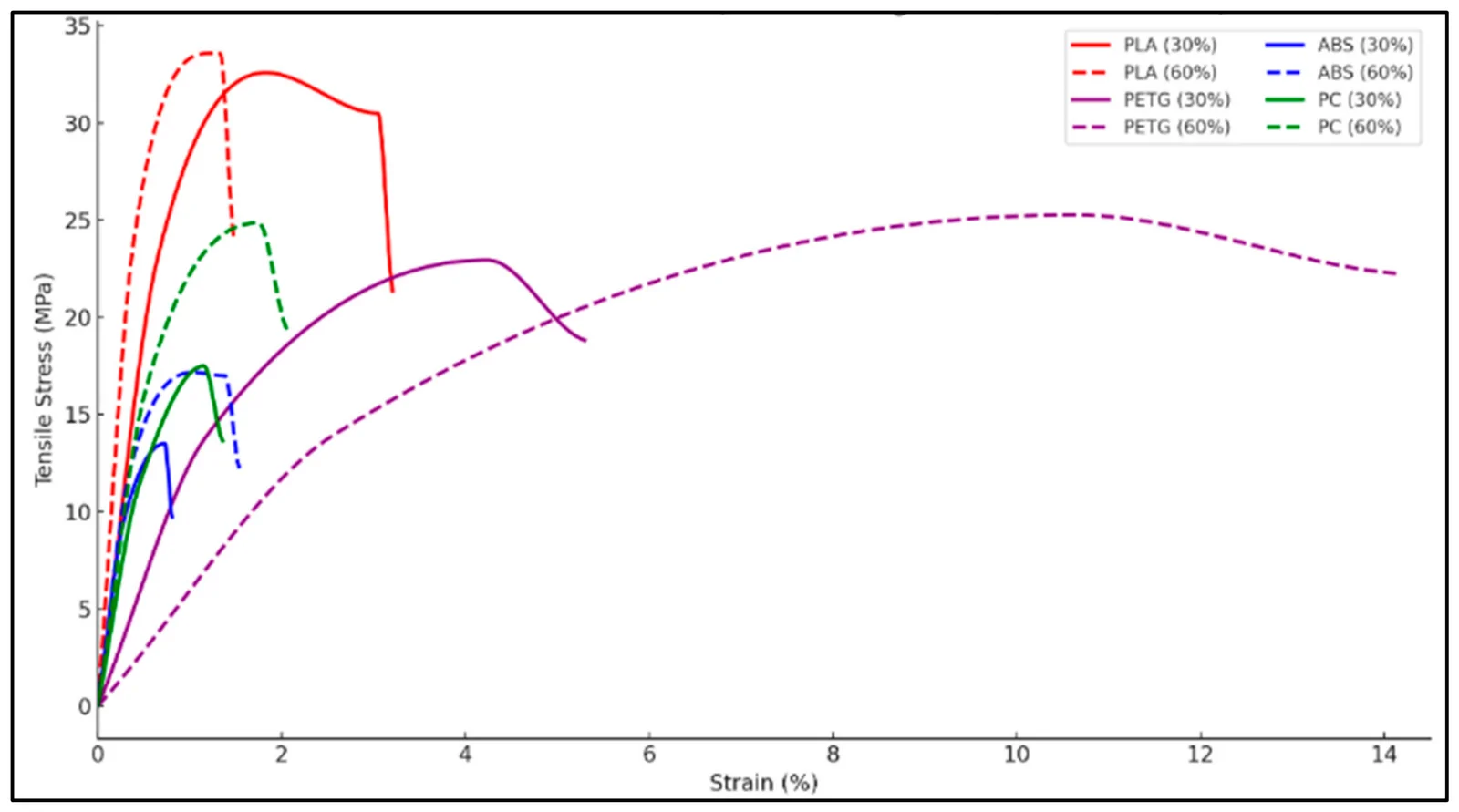
Stress–strain curves of test specimens.

Tensile test results of specimens produced with different infill densities are presented in Table 5 and Figure 4. In the study, increasing the infill density from 30% to 60% resulted in an increase in tensile stress values for all materials. This can be attributed to the decrease in porosity and strengthening of internal bonds at higher infill densities, resulting in a stronger load-bearing section. PLA achieved the highest tensile stress values, reaching 33.57 MPa at a 60% infill density. PC followed at 24.86 MPa, and both materials were found to be suitable for applications requiring high rigidity and tensile strength. While PETG and ABS recorded lower tensile stress values, they exhibited competitive performance, particularly in applications requiring impact resistance or flexibility. In terms of ductility, PETG had the highest elongation, particularly at 60% infill (14.18%). ABS and PC exhibited normal elongation, while PLA had the lowest elongation values, indicating that despite its high strength, it exhibited relatively brittle behavior. Comparing the effects of infill, the recovery rate varied depending on the material. For ABS, the tensile stress increased from 13.50 MPa to 16.99 MPa, while its elongation increased from 0.81% to 1.54%; for PC, the tensile stress increased from 17.49 MPa to 24.86 MPa, while its elongation increased from 1.36% to 2.06%. For PLA, the tensile stress increased from 30.50 MPa to 33.57 MPa, but its elongation decreased from 3.21% to 1.48%. In PETG, the tensile stress increased from 22.95 MPa to 25.27 MPa, while the elongation increased significantly from 5.30% to 14.18%. In addition, the standard deviation (0.68–1.68 MPa) and coefficient of variation (4.97–5.04%) values for the tensile test indicate acceptable data dispersion and consistent experimental repeatability. The relatively low standard deviation values show that the measurements were reasonably clustered around the mean, while the coefficient of variation values of approximately 5% confirm satisfactory measurement consistency and reliability.

In general, material type was the most decisive factor in tensile performance; PLA and PC stood out for their high strength, while PETG yielded the best ductility results. The infill density also played a significant role in tensile performance, generally increasing strength, but its effect on ductility varied by material; ABS and PETG increased ductility, while PLA decreased it. While hardness increased with higher infill density for all investigated materials, tensile modulus exhibited material-dependent behavior. PLA showed a substantial increase in modulus with increasing infill density, indicating that the enhanced structural continuity contributed to both higher stiffness and surface hardness. ABS exhibited nearly identical modulus values at both infill densities, suggesting that the increase in hardness had only a limited influence on its elastic response. PC exhibited only a slight decrease in modulus, which may be attributed to experimental variability and does not indicate a significant change in elastic behavior. In contrast, PETG showed a noticeable decrease in modulus despite the increase in hardness. This behavior has been attributed to differences in the deformation mechanisms of FDM-printed polymers. Although increasing infill density improves hardness and tensile stress by reducing internal porosity, it may also promote greater elastic-plastic deformation during tensile loading in relatively ductile materials, thereby reducing the initial elastic slope from which tensile modulus is determined.

To compare the results obtained, the tensile properties were compared with those reported for fully dense (100% infill) and high-density FDM specimens available in the literature. Previous studies consistently demonstrate that increasing infill density improves mechanical performance by reducing internal porosity and enhancing interlayer bonding. For example, it has been reported that PLA specimens printed with 100% infill exhibited the highest tensile strength among specimens manufactured with 25%, 50%, 75%, and 100% infill densities [5]. Similarly, increasing infill density was reported to significantly enhance the tensile performance of PLA, ABS, and PETG components, with PLA exhibiting an increase of 1173 N between 20% and 80% infill [17]. Additionally, infill density was identified as one of the most influential process parameters affecting the tensile performance of FDM-printed PLA, ABS, and PETG, while PLA exhibited the highest tensile strength among the investigated materials [18]. Compared with these fully dense specimens, the tensile strengths obtained in the present study at 60% infill density are naturally lower due to the presence of a larger internal porous volume. Nevertheless, the measured values follow the same increasing trend reported in the literature, confirming that increasing infill density (from 30% to 60% infill) improves load transfer efficiency and reduces stress concentration. Therefore, the present findings are in good agreement with previous studies. These findings indicate that 60% infill density provides a practical compromise between mechanical performance, material consumption, and manufacturing time, making it particularly attractive for lightweight engineering applications where fully dense structures are not required.

Two-way ANOVA analysis of tensile strength in Table 6 revealed that both material type and infill density had statistically significant effects on the tensile strength of FDM-printed specimens (*p* < 0.001). Among the investigated factors, material type had the most dominant influence, explaining 83.91% of the total variance in tensile strength. This indicates that the structural and mechanical differences between engineering polymers are the primary determinants of tensile performance. Although infill density had a comparatively lower contribution (Pc = 11.59%), it was still found to be statistically significant (*p* < 0.001), highlighting that this manufacturing parameter should also be considered when aiming to optimize mechanical properties. Moreover, the interaction effect between material type and infill density was statistically significant (*p* = 0.0231), although its contribution was smaller (Pc = 1.98%). This finding suggests that the influence of infill density on tensile strength varies depending on the type of material used.

### 3.3. The Charpy Impact Test

**Figure 5:**
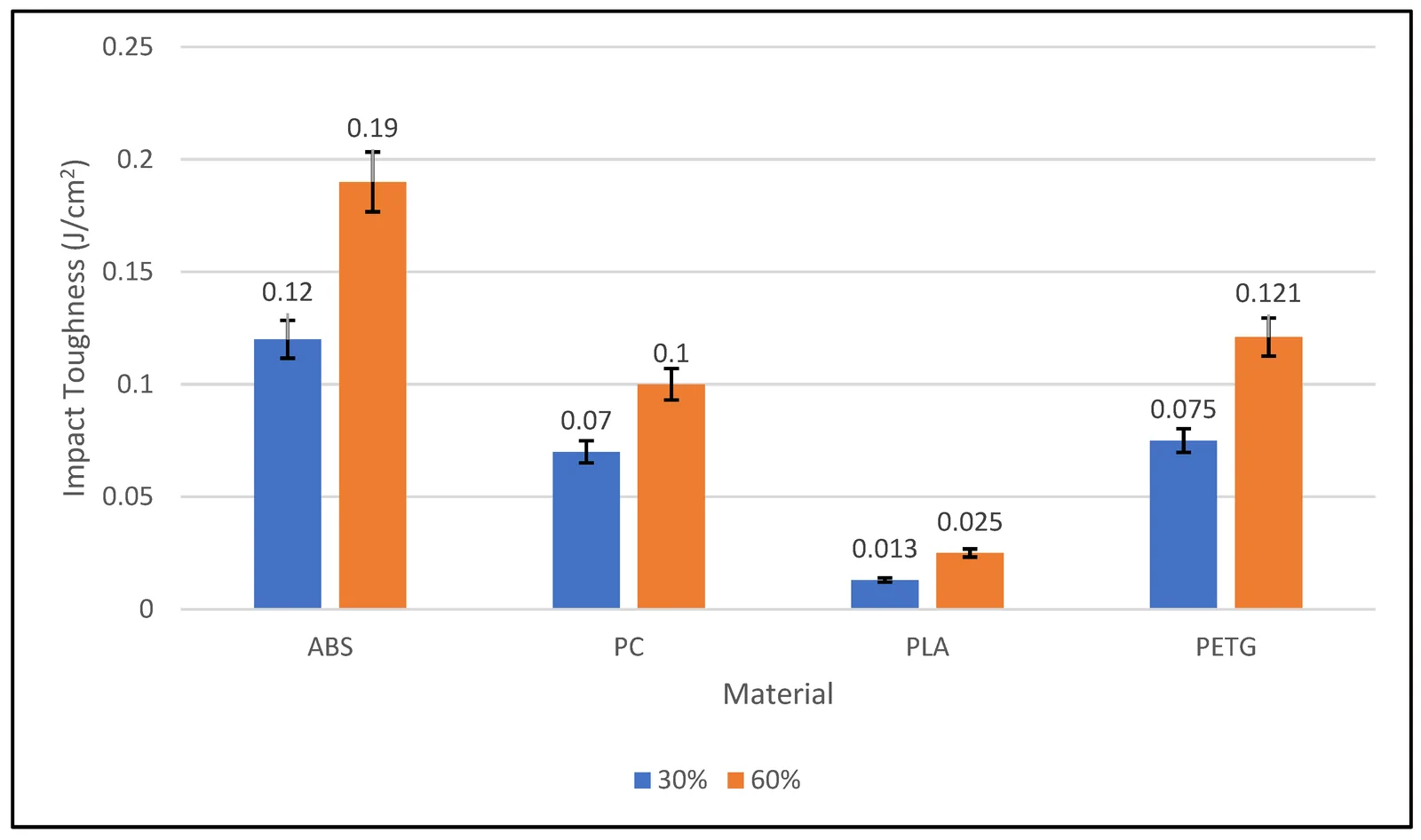
The graph of impact test.

Charpy impact test results of the specimens are given in Table 7 and Figure 5. According to the Charpy impact test results, the highest impact energy and impact toughness values were obtained for the PETG specimen with a 60% infill density (0.96 J and 0.121 J/cm^2^). This indicates that PETG increases its structural integrity and impact absorption capacity. Similarly, the impact strength of the ABS specimens increased with increasing infill density; the impact energy increased from 0.46 J for 30% ABS to 0.71 J for 60% ABS, and the impact toughness increased from 0.120 J/cm^2^ to 0.190 J/cm^2^. This increase was more limited in the PC specimens, with the impact energy increasing from 0.26 J at 30% fill to 0.38 J at 60% fill, and impact toughness increasing from 0.070 to 0.100 J/cm^2^. On the other hand, PLA had the lowest impact toughness values at both 30% and 60% fill (0.013 and 0.025 J/cm^2^, respectively). This clearly demonstrates PLA’s brittle nature and low impact strength.

For the Charpy impact test, the standard deviation (0.0009–0.0133 J/cm^2^) and coefficient of variation (6.92–7.20%) values indicate acceptable data dispersion and consistent experimental repeatability. The relatively low standard deviation values show that the measurements were reasonably clustered around the mean, while the coefficient of variation values of approximately 7% confirm satisfactory measurement consistency and reliability.

In general, although increasing the infill density increases impact resistance in all materials, the material’s internal structure and its susceptibility to plastic deformation are the primary factors determining this increase. PETG and ABS stand out as the most effective materials in terms of energy absorption capacity under impact loads.

Two-factor ANOVA analysis in Table 8 showed that both material type and infill density had significant effects on impact toughness, and that these two factors interacted. Material type was the most dominant factor (86.72%), while infill density (7.82%) and the interaction (3.89%) were also found to be statistically significant. The low error rate of 1.57% confirmed the consistency and reliability of the tests.

### 3.4. SEM-EDS Analysis

The SEM analysis images were taken from the surface at 1000× and 5000× magnification with a 10 µm scale.

**Figure 6:**
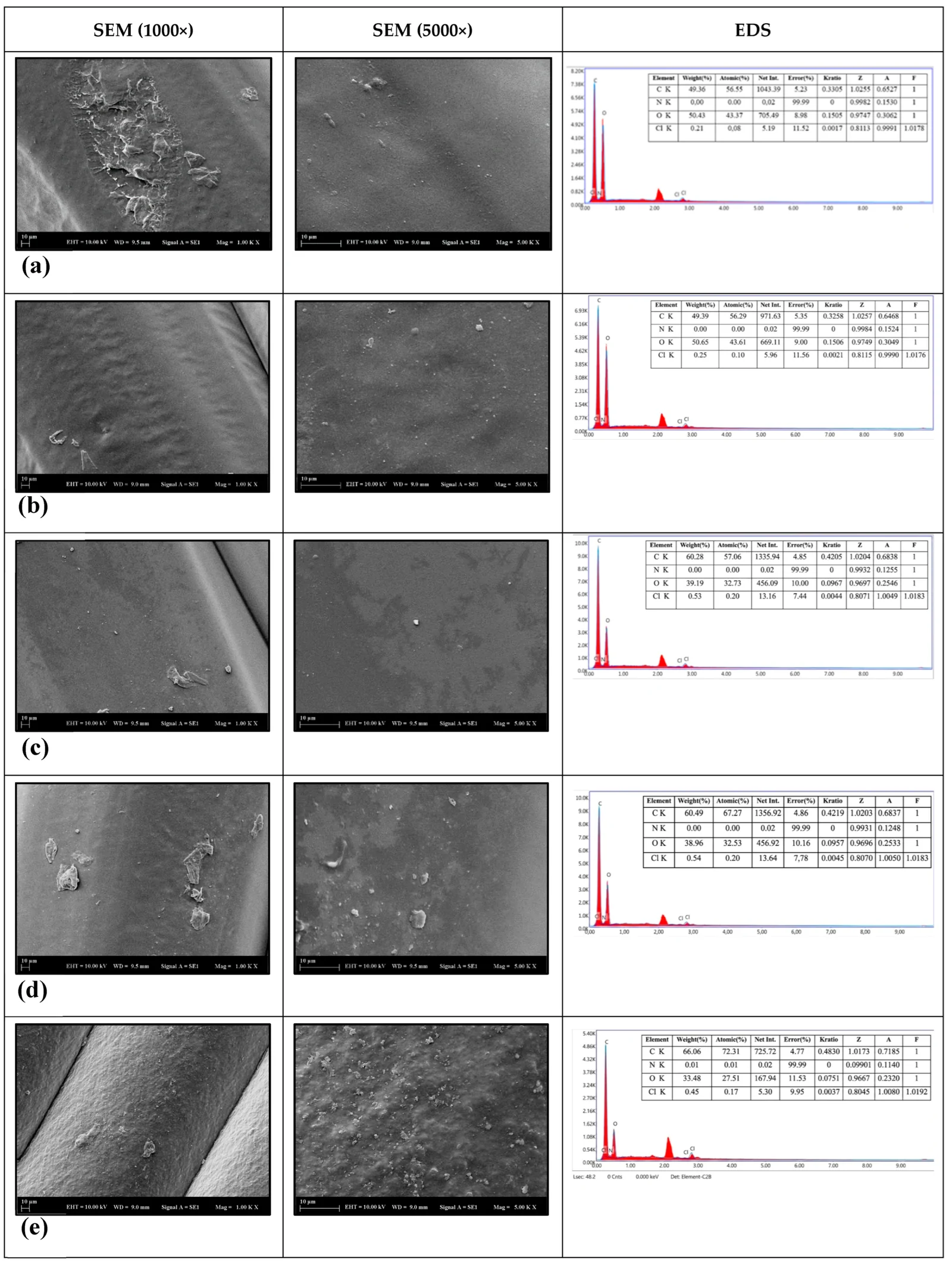

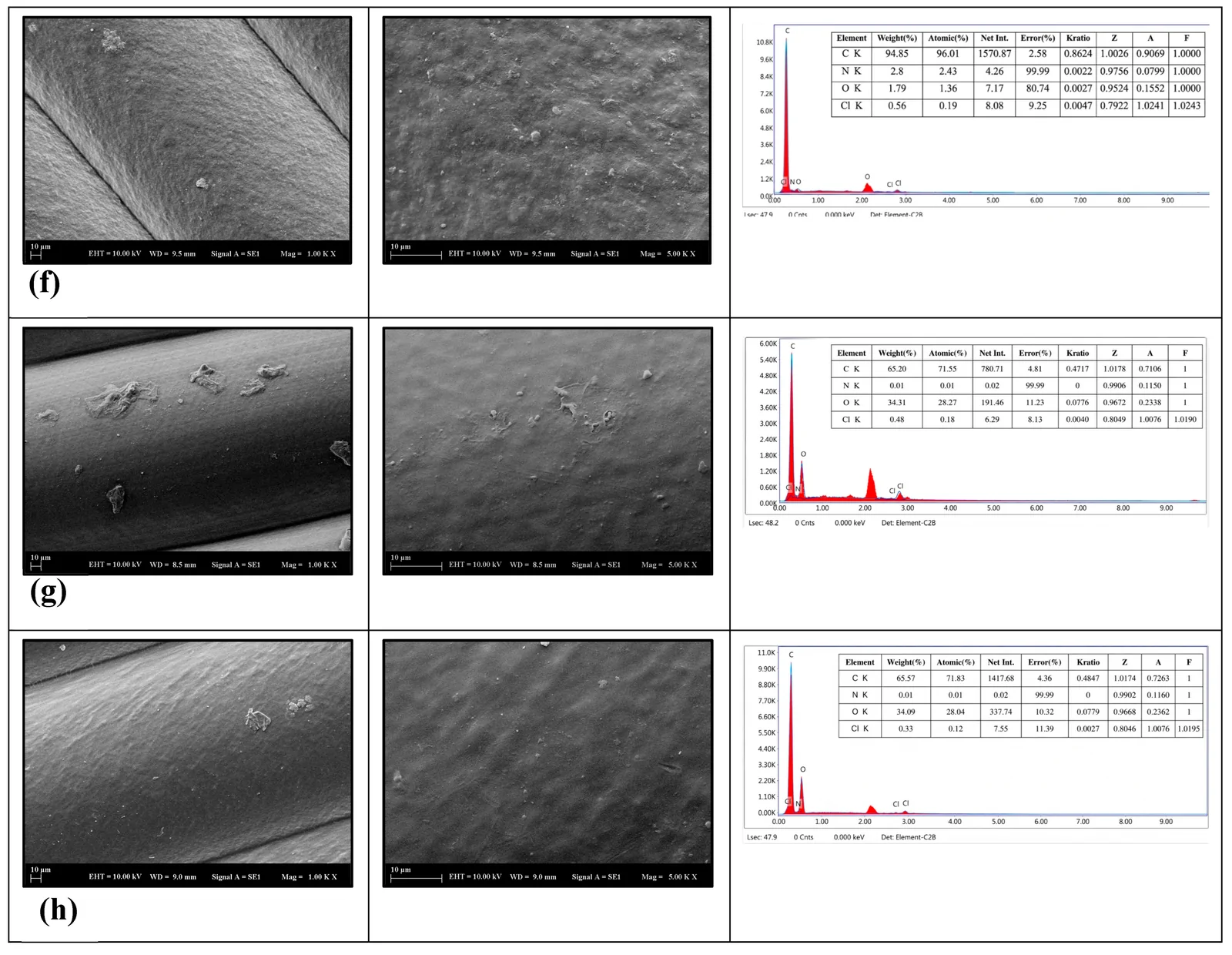
SEM-EDS analysis of test specimens (**a**) %30 PLA, (**b**) %60 PLA, (**c**) %30 PETG, (**d**) %60 PETG, (**e**) %30 ABS, (**f**) %60 ABS, (**g**) %30 PC, (**h**) %60 PC.

In Figure 6a, the SEM image of PLA at 30% infill density reveals crack-like structures and irregular voids on the surface. The microstructure shows signs of weak bonding. In Figure 6b, PLA at 60% infill density exhibits a more uniform and dense structure, with reduced porosity. The surface appears smoother, and microcracks are reduced. The more homogeneous and compact structure of PLA at 60% infill density contributes to its higher mechanical tensile strength (33.57 MPa). However, due to its low ductility, small cracks in the microstructure result in lower impact performance (minimum 0.013 J/cm^2^). In Figure 6c, the SEM image of PETG at 30% infill density shows some particle inclusions, rough areas on the surface, and void structures. In Figure 6d, more pronounced adhesion marks and fibrous structures are noticeable on PETG at 60% infill density. Surface porosity has decreased, resulting in a more compact structure. PETG exhibits better material homogeneity at 60% infill density. The regular fiber structure and reduced voids in the SEM images directly correlate with PETG’s highest impact strength (0.121 J/cm^2^) at 60% infill density. This supports its suitability for load-bearing structures. In the SEM image of ABS at 30% infill density in Figure 6e, granular particles and some voids are seen on the surface, where filament layers are clearly visible. In Figure 6f, ABS at 60% infill density exhibits a more tightly bonded filament structure and a homogeneous and dense surface appearance. However, some particle clusters are noticeable. ABS performs quite well in terms of impact resistance. This can be explained by the strong filament bonds seen in SEM images and the decreasing voids with infill density. However, the relatively low tensile strength (16.99 MPa) and moderate ductility in mechanical tests also indicate that this structure is prone to brittleness. PC at the 30% infill density in Figure 6g exhibits fibrous inclusions and an irregular surface texture throughout the filament structure. A denser surface, regular filament bonding, and micro-particle inclusions were observed in the 60% infill density at PC in Figure 6h. PC demonstrates that it is the most structurally resistant material (tensile strength 24.86 MPa). SEM images support this conclusion, demonstrating a structure with better bonding and fewer voids at higher infill density. Furthermore, PC’s moderate impact strength (0.100 J/cm^2^) and high surface order make it a preferred choice for structural parts.

In conclusion, SEM analyses clearly reveal the microstructure of the materials, which varies with the infill density. Particularly at higher infill densities, reduced porosity within the material, better bonding of filament layers, and reduced particle irregularities all contributed to improved mechanical properties such as tensile strength, impact strength, and hardness. Thus, a strong micromechanical relationship was established between the infill density and the material type.

The EDS analyses in Figure 6 confirmed that the printed specimens were primarily composed of carbon and oxygen, consistent with the characteristic elemental composition of the investigated polymers. Small variations in the measured carbon and oxygen contents were observed among specimens with different infill densities. However, these differences should not be interpreted as changes in the intrinsic chemical composition of the materials, since infill density modifies the internal structure rather than the polymer chemistry. Instead, the observed variations are attributed to differences in fracture morphology, exposed surface features, local porosity, and the specific regions analyzed by EDS. Therefore, similar to the SEM images, the EDS results support the microstructural differences associated with infill density rather than indicating changes in the surface chemistry of the printed materials.

## 4. Conclusions

In this study, the mechanical and microstructural properties of ABS, PC, PLA, and PETG materials produced by the FDM method at 30% and 60% infill densities were comparatively investigated using tensile, Charpy impact, hardness, and SEM-EDS analyses. The results clearly showed that both material type and infill density have a significant effect on overall mechanical performance. However, material type was determined to be the dominant parameter in all statistical evaluations.

Hardness results showed that Shore D hardness values increased with increasing infill density in all materials due to decreased porosity and improved internal bonding. PLA exhibited the highest hardness values at both infill densities, confirming its inherently rigid and brittle structure. ABS, on the other hand, had the lowest hardness values. According to the two-way ANOVA analysis, 59.34% of the variance in hardness was due to material type and 27.87% was due to infill density, and the interaction effect was statistically significant. Consistent with the literature, these findings reveal that infill optimization can increase surface resistance, but hardness behavior is primarily determined by the internal structure of the polymer [13,14,15,16,17,18].

Tensile test results showed that increasing the infill density from 30% to 60% increased the tensile strength in all materials. This increase is related to the reduction in internal voids and the expansion of the load-bearing cross-sectional area. PLA showed the highest tensile strength at 60% infill with 33.57 MPa, followed by PC with 24.86 MPa. These results indicate that these materials are suitable for applications requiring high rigidity and structural strength. PETG exhibited the highest ductility at 60% infill with an elongation of 14.18%. PLA, despite its high strength, revealed its brittle character with a low elongation value. The tensile modulus exhibited a material-dependent response, with PLA showing the highest modulus at 60% infill, whereas ABS and PC changed only slightly and PETG exhibited a lower modulus. The tensile strain at break also depended on the material type. PETG exhibited the highest strain at break, indicating superior ductility, whereas PLA showed the lowest values, confirming its relatively brittle behavior despite its high tensile strength. ANOVA results showed that 83.91% of the variance in tensile performance was due to the material type. Infill density was statistically significant but had a secondary effect. As indicated in the literature, the interaction between material type and infill density also showed that the mechanical improvement provided by the increase in infill is material-dependent [5,17,20].

Charpy impact test results revealed that PETG (0.121 J/cm^2^ at 60% infill) and ABS exhibited superior energy absorption capacity. This indicates that these materials are more suitable for applications subjected to impact loads. PLA, on the other hand, showed the lowest impact strength values at both infill densities, confirming its brittle fracture behavior. Although increasing the infill density improved impact strength in all materials, according to ANOVA analysis, 86.72% of the variance was due to the material type. This result shows that impact behavior largely depends on the intrinsic properties of the polymer.

SEM analyses provided significant microstructural evidence supporting the mechanical findings, which are consistent with the literature [16]. High infill density contributed to reduced porosity, improved interfilament fusion, and a more homogeneous internal structure in all materials. In PLA specimens, a reduction in microcracks and the formation of a denser bonding structure were observed at 60% infill; this explains the increase in tensile strength while revealing that brittle behavior under impact persisted. In PETG, a more regular fibrous structure and improved interlayer adhesion were observed; this was directly correlated with superior impact performance. In ABS and PC specimens, it was determined that filament bonding was strengthened and void formation was reduced to 60% infill, supporting improvements in tensile and impact strength.

In general, this study revealed that increasing the infill density improves mechanical properties by enhancing internal structural integrity; however, the primary determinant of performance is the type of material. ABS and PC demonstrated promising characteristics for engineering applications requiring high strength and impact resistance, whereas PLA and PETG exhibited favorable properties for applications where lightweight design and environmentally friendly materials are desirable. Additionally, although 60% infill density requires more material and longer printing time than 30% infill density, it provides substantially improved tensile strength, impact resistance, hardness, and microstructural integrity due to reduced internal porosity and stronger interlayer bonding. Consequently, 60% infill density represents a practical compromise for engineering applications requiring enhanced mechanical reliability without the excessive material consumption and printing time associated with fully dense (100% infill) structures. Accordingly, 30% infill density may be preferred for non-structural or prototype components where lightweight design and rapid fabrication are prioritized, whereas 60% infill is more suitable for semi-structural and load-bearing applications requiring improved mechanical performance. These findings provide a comprehensive experimental and statistically based framework for optimizing material selection and infill density in FDM manufacturing processes. Furthermore, they provide an initial basis for material selection and process optimization in additive manufacturing, while their applicability to specific engineering fields, such as automotive, biomedical, and structural applications, should be further validated through application-specific studies. It is emphasized that the present findings are limited to the experimental conditions investigated in this study. Since the effects of aging, moisture exposure, thermal conditions, and long-term service environments were not evaluated, these findings should be considered a preliminary assessment. Therefore, further application-specific studies are required before generalizing the present results to practical engineering applications.

## Figures and Tables

**Table 1:** Physical and mechanical properties of ABS, PC, PLA, and PETG filaments.

Properties	ABS	PC	PLA	PETG
Density (g/cm^3^)	1.03–1.07	1.18–1.22	1.24–1.30	1.23–1.27
Melting temperature (°C)	210–250	260–310	180–220	220–260
Tensile strength (MPa)	30–50	55–75	50–70	40–55
Flexural strength (MPa)	60–90	90–120	80–110	70–100
Elastic modulus (GPa)	1.8–2.5	2.0–2.5	3.0–4.0	1.9–2.4
Impact strength (J/m)	10–30	30–60	2–5	5–15
Hardness (Shore D)	70–80	80–85	75–80	72–78
Heat deflection temp. (°C, HDT)	80–100	120–140	50–60	70–80

**Table 2:** FDM process parameters used in this study.

Parameter	Values
Layer height	0.20 mm
Printing speed	60 mm s^−1^
Infill density	30% and 60%
Infill pattern	Grid
Nozzle diameter	0.4 mm
Ambient temperature	25 °C (room temperature)
Relative humidity	40%
Support structures	Not used
PLA nozzle and build plate temperatures	210 °C and 60 °C
ABS nozzle and build plate temperatures	230 °C and 100 °C
PETG nozzle and build plate temperatures	240 °C and 70 °C
PC nozzle and build plate temperatures	280 °C and 120 °C

**Table 3:** Hardness test results.

Specimens	Hardness (Shore D)	Standard Deviation (Shore D)	Coefficient of Variation (%)
30% ABS	64.55	0.17	0.26
60% ABS	72.05	0.41	0.57
30% PC	66.16	0.41	0.62
60% PC	69.63	0.23	0.33
30% PLA	74.00	0.48	0.65
60% PLA	75.00	0.27	0.36
30% PETG	68.00	0.62	0.91
60% PETG	70.00	0.32	0.46

**Table 4:** ANOVA analysis for hardness.

Factor	DoF	F	*p* Value	Pc (%)
Material type (A)	3	247.84	1.28 × 10^−13^	59.34
Infill density (B)	1	349.23	2.72 × 10^−12^	27.87
A (Material type) × B (Infill density)	3	48.1	3.14 × 10^−8^	11.51
Error	16	-	-	1.28

**Table 5:** Tensile test results.

Specimens	Tensile Force (N)	Tensile Stress (MPa)	Tensile Modulus (GPa)	Strain at Break (%)	Strain (%)	Tensile Stress Standard Deviation (MPa)	Tensile Stress Coefficient of Variation (%)
30% ABS	823.33	13.50	11.74	0.81	1.15	0.68 MPa	5.04
60% ABS	1036.66	16.99	11.72	1.54	1.45	0.85 MPa	5.00
30% PC	1066.66	17.49	15.90	1.36	1.10	0.87 MPa	4.97
60% PC	1516.66	24.86	14.62	2.06	1.70	1.24 MPa	4.99
30% PLA	1860.66	30.50	16.94	3.21	1.80	1.53 MPa	5.02
60% PLA	2047.55	33.57	26.86	1.48	1.25	1.68 MPa	5.00
30% PETG	1399.93	22.95	5.40	5.30	4.25	1.15 MPa	5.01
60% PETG	1541.64	25.27	2.41	14.18	10.50	1.26 MPa	4.99

**Table 6:** ANOVA analysis of tensile strength.

Factor	DoF	F	*p* Value	Pc (%)
Material type (A)	3	177.03	1.76 × 10^−12^	83.91
Infill density (B)	1	73.34	2.27 × 10^−7^	11.59
A (Material type) × B (Infill density)	3	4.18	2.31 × 10^−2^	1.98
Error	16	-	-	2.53

**Table 7:** Charpy impact test results.

Specimens	Impact Energy (J)	Impact Toughness (J/cm^2^)	Standard Deviation for Impact Toughness (J/cm^2^)	Coefficient of Variation Impact Toughness (%)
30% ABS	0.46	0.120	0.0084	7.00
60% ABS	0.71	0.190	0.0133	7.00
30% PC	0.26	0.070	0.0049	7.00
60% PC	0.38	0.100	0.0070	7.00
30% PLA	0.10	0.013	0.0009	6.92
60% PLA	0.20	0.025	0.0018	7.20
30% PETG	0.60	0.075	0.0053	7.07
60% PETG	0.96	0.121	0.0085	7.02

**Table 8:** ANOVA analysis for the Charpy impact test.

Factor	DoF	F	*p* Value	Pc (%)
Material type (A)	1	442.59	2.74× 10^−8^	86.72
Infill density (B)	1	39.92	2.28 × 10^−4^	7.82
A (Material type) × B (Infill density)	1	19.84	2.13 × 10^−3^	3.89
Error	8	-	-	1.57

## Data Availability

The original contributions presented in this study are included in the article. Further inquiries can be directed to the corresponding author.
